# A New Approach to LCA Evaluation of Lamb Meat Production in Two Different Breeding Systems in Northern Italy

**DOI:** 10.3389/fvets.2020.00651

**Published:** 2020-09-28

**Authors:** Andreas Geß, Irene Viola, Silvia Miretti, Elisabetta Macchi, Giovanni Perona, Luca Battaglini, Mario Baratta

**Affiliations:** ^1^Department of Life Cycle Engineering, Institute for Acoustics and Building Physics, University of Stuttgart, Stuttgart, Germany; ^2^Department of Veterinary Science, University of Turin, Grugliasco, Italy; ^3^Department of Veterinary Science, University of Turin, Turin, Italy; ^4^Department of Agricultural, Forest and Food Sciences (DISAFA), University of Turin, Grugliasco, Italy

**Keywords:** lamb, breeding system, LCA, global warming potential, acidification potential, eutrophication potential, animal welfare, Italy

## Abstract

Lamb meat production provides vital landscape-management and ecosystem services; however, ruminant farming produces a considerable share of the world's greenhouse gas emissions. To measure and compare the advantages and disadvantages of the intensification of livestock farming, an integrative analysis was conducted in this study by combining environmental impact analysis and animal welfare assessment. This approach is the first of its kind and is the innovative aspect of this paper. The methodology of Life Cycle Assessment (LCA) entails the holistic analysis of various impact categories and the associated emission quantities of products, services, and resources over their life cycle, including resource extraction and processing, production processes, transport, usage, and the end of life. The outlines of LCA are standardized in DIN EN ISO 14040/14044. To assess the environmental impacts of the production of lamb meat in northern Italy, two case studies were undertaken using the LCA software GaBi. The analysis is based on primary data from two sheep-breeding systems (semi-extensive and semi-intensive in alpine and continental bioregions, respectively) combined with inventory data from the GaBi database and data from the literature. The assessment was conducted for the functional unit of 1 kg of lamb meat and focuses on the impact categories global warming potential, acidification potential, and eutrophication potential. For an overall evaluation of the supply chain, we have also considered a parameter indicating animal welfare, in keeping with consumer concerns, employing an analysis of chronic stress as shown by cortisol accumulation. The goal is to derive models and recommendations for an efficient, more sustainable use of resources without compromising animal welfare, meat quality, and competitiveness. The aim of this study is to provide a standard for individualized sustainability analyses for European lamb production systems in the future. From the LCA perspective, the more intensive case-study farm showed a lower impact in global impact factors and a higher impact in local impact categories in comparison with the more extensively run farm that was studied. From the animal welfare perspective, lower amounts of the stress hormone cortisol were found on the extensively managed case-study farm.

## Introduction

The increasing concern in society regarding the consumption of products of animal origin has drawn attention to the need of understanding how the production process could be carried out in a sustainable manner (1). Within sheep farming there are extrinsic and intrinsic factors that could affect the acceptability of lamb meat in accordance with production systems that can be characterized to varying degrees as either extensive or intensive (2). In Europe, these farming systems for meat production are complex and diverse. They reflect different local environmental conditions and cultural practices and thus give rise to different husbandry approaches. These local conditions determine, to a large extent, the choice of breeds used, housing conditions, diets, levels of intensification, liveweight at slaughter and, ultimately, and local market requirements (3). This variability can be regarded as an advantage for European lamb producers because it offers opportunities for change or diversification (4).

Within the food production sector, the meat industry claims the lion's share of the carbon footprint. Livestock farming produces almost 80% of all emissions from the agricultural industry sector and about 35–40 % of all global anthropogenic methane emissions (5). Intensive farming has therefore been promoted to increase productivity and thereby reduce relative carbon emissions (6). This, however, contributes to local effects, such as eutrophication and acidification, while the positive effects of extensive management practices are often not accounted for (7). In central Europe, however, extensive animal farming has played—and still plays—a vital role in the creation and preservation of the characteristic cultural landscape, and the endemic flora and fauna depends on the herds of livestock passing by (8). Due to industrialization and intensification, the number of extensive farming systems and thus the total number of grazed pastures in the Mediterranean region has decreased in recent decades (9).

The assessment of the emissions of agricultural production systems can be conducted using the Life Cycle Assessment (LCA) method (10). LCA evaluates the impacts of products during their whole life cycle, employing standardized methods and impact categories (11, 12). All impacts are calculated in relation to the functional unit, which in the case of this study is 1 kg of lamb meat. The assessment of agricultural systems using LCA has been and remains a special challenge because of an insufficiency of available data and because most agricultural processes have multiple indistinct inputs and outputs. For these reasons, it is very difficult to quantify the production systems. In addition, results may vary strongly depending, for example, on breeds, local factors, or seasonal variability in the respective case study (13).

Nevertheless, sheep farming in most European countries is mainly characterized by extensive systems based on the use of grasslands and pastures that are often located in mountainous and marginal areas (14).

Pasture-based sheep farming—which is particularly widespread in Mediterranean areas—performs a variety of functions (including, for example, the promotion of biodiversity and landscape conservation). The need to consider these functions as part of those systems is well known. These practices vary greatly from those employed in the intensive production systems, which are more typical of continental systems (4). In the latter systems, increased productivity and efficiency in lamb production is a key factor in increasing the competitiveness of the sheep meat industry. On the other hand, the adoption of rearing practices with low feeding intensities takes into consideration the needs of endangered breeds, the conservation and appreciation of which are closely linked to the preservation and development of more ecologically sustainable livestock production systems. This is the case with several Italian rare breeds for which feeding strategies are based on the exclusive utilization of grasslands (15).

The welfare of food animals is a growing concern. Farming of animals under human care is no longer seen as merely a means of food production but also as an ethical concern that also has economic implications (16). Therefore, even if farmers and producers are often reluctant to accept some policies intended to improve animal welfare, claiming it will increase production costs and reduce production efficiency, a sustainability assessment of the supply chain must begin to understand different parameters that interpret the concept of sustainability in a more extensive way according to the preferences and indications of the consumer.

Cortisol is a glucocorticoid produced by the adrenal cortex in response to adrenocorticotropic hormone (ACTH) secretion. It is considered an indicator of the body's hormonal responses to stress and is regulated by the HPA-axis. Cortisol can be measured in blood, urine, saliva, and recently also in hair samples. Hair analysis has increasingly been used as a non-invasive method to obtain information on long-term HPA-axis activity for the evaluation of chronic stress with a negligible influence of acute stress (17). The choice of this method is particularly suitable for the analysis of an extensive supply chain where contact with animals is less and the evaluation of stress could be influenced by the same manipulation for the collection of biological samples.

We report here on the analysis of two different breeding systems in lamb meat production (semi-extensive and semi-intensive) in north-western Italy, which were evaluated according to an LCA model that took into account the different bioregions; at the same time, attention was also paid to the considerable consumer concern regarding the management of animal welfare throughout the process supply chain. Animal welfare was assessed through the accumulation of cortisol in the fleece of the animals throughout the breeding period. The accumulation of this hormone is considered to be a significant parameter for the evaluation of the chronic stress to which the animal has been subjected (18). The innovative aspect of this paper lies in the combination of animal welfare analysis and the LCA method for sheep farming. In this way, the lack of an assessment of animal treatment in LCA is complemented by analyzing environmental impacts, to provide a more holistic insight into different approaches to of sheep farm management.

## Materials and Methods

### Farming Systems

#### Continental Semi-Intensive System

In the continental bioregion, located in the Po Valley, in Turin (in northwestern Italy), Biellese lambs were raised at CISRA, the Teaching Animal Farm of the Department of Veterinary Science at the University of Turin. The production system used for the Biellese breed is semi-intensive in character. In this semi-intensive system, the Biellese lamb flocks were released to graze outside in the autumn-to-winter season (period of investigation); in this system, 28 Biellese lambs were bred from birth to 4 to 5 months of age and then brought to the slaughterhouse. The teaching farm has a prolificacy rate of about 120%, and 50 lambs are born every year. The birthweight is 5 ± 0.4 kg. The lambs consume about 500 gr of milk per day. They are weaned at 60 days when their weight is around 18 ± 2.5 kg. After weaning, they consume approximately 150 gr of concentrate per day and as much hay as they want until they are slaughtered (at an average age of 4.5 months). The average daily weight increase was 180 ± 30 gr. The flock spends 6 months (from October to March) in the stable, free to move with an external paddock, and 6 months (from April to September) in a fenced grazing area of measuring four ha. The number of animals per ha is 21. The sheep are fed hay and pelleted concentrate (see Supplementary Data, Table A). Each animal was fed 0.5 to 1.5 kg/day of concentrate during the breeding periods (March and September).

#### Semi-Extensive System

In the alpine bioregion, located in Val Maira (CN) in the western Alps at an altitude of 1,700–2,000 m.a.s.l., during the summer season, Sambucana lambs are kept in a permanent grazing system. The production system for 32 Sambucana lambs that was analyzed was thus based on a natural alpine pasture. The prolificity rate was 150%, and the number of lambs born per year was 350 with an average birth weight of 4 ± 0.7 kg. They were all born in May and grazed from dawn until evening; they were surrounded overnight by electric fencing and were further protected from predators by guard dogs. The lambs' estimated milk consumption was about 400 gr of maternal milk per day until weaning (60 days); then they grazed in the alpine pasture until 4 to 5 months of age, and they were slaughtered in September at a weight of 30–35 kg. The daily weight gain was 200 ± 25 gr. The pasture stocking rate was 5 adult heads per hectare. They were fed at pasture, apart from the stabled period after birth, consuming maternal milk and some hay. A pelleted concentrate (see Supplementary Data, Table B) was supplied to lactating ewes (500 gr/day) and to lambs born in winter (250 gr/day).

### Methodology

#### Experimental Design

##### Wool sample collection

The timeline of the experimental design provided for the collection of wool samples every 30 days, from the lambs' first month of life (T1) to the age of 4 months (T4). The procedures have been authorized by the Ethics and Animal Welfare Commission (n 1865 of December 7, 2017) of the University of Turin.

Wool samples were collected using a shave and re-shave method. Old wool was removed prior to the start of the experiment, which was carried out within 15 day after lambing (giving birth) (18). Wool samples were obtained from the posterior vertex region of the neck between the cisterna magna and scapular bones (19). Wool was carefully shaved with available pet-grooming clippers without damaging the skin or hair follicles. Approximately 10 cm^2^ was shaved in order to obtain sufficient wool for lab analysis.

The wool samples were stored immediately in aluminum foil and then in labeled paper envelopes at room temperature until analysis (18, 19).

### Hormone Analysis

Prior to extraction, 250 mg of each wool sample was washed with 5 ml of isopropanol (Sigma Aldrich, IT), mixed for 3 min in a rotator at room temperature for 3 min per wash, and dried in a fume hood for 2 days as suggested by Davenport et al. (20). Wool cortisol extraction was performed according to the method described by Koren et al. (21), with some modifications. The dried wool was cut into 1–3 mm-long fragments with scissors, two 60 mg aliquots were put into a 5 ml glass vial, and 3 ml of methanol (Sigma Aldrich, IT) was added. The vials were incubated at 37°C under an airstream suction hood for 18 h and centrifuged for 15 min at 2,500 rpm. The supernatant collected in glass vials was placed under an airstream suction hood at 37°C until it had dried completely. Extracts were stored in a frozen state until analysis.

Extracted samples were reconstituted in duplicate with 250 μL of ImmunoAssay Buffer (IAB) before the quantification of cortisol in the wool. Wool cortisol levels were determined using a commercial AlphaLISA Assay Kit (Cortisol AlphaLISA Kit—PerkinElmer, USA) according to the manufacturer's specifications. The intra-assay and inter-assay coefficients of variation were 3 and 4%, respectively. The analytical sensitivity (Lower Detection Limit, LDL) of the method is 177 pg/ml and it shows the following cross-reactivity: 21- deoxycortisol 9%, prednisolone 5%, cortisone, and corticosterone 1%.

#### LCA Analysis

**Figure 1:**
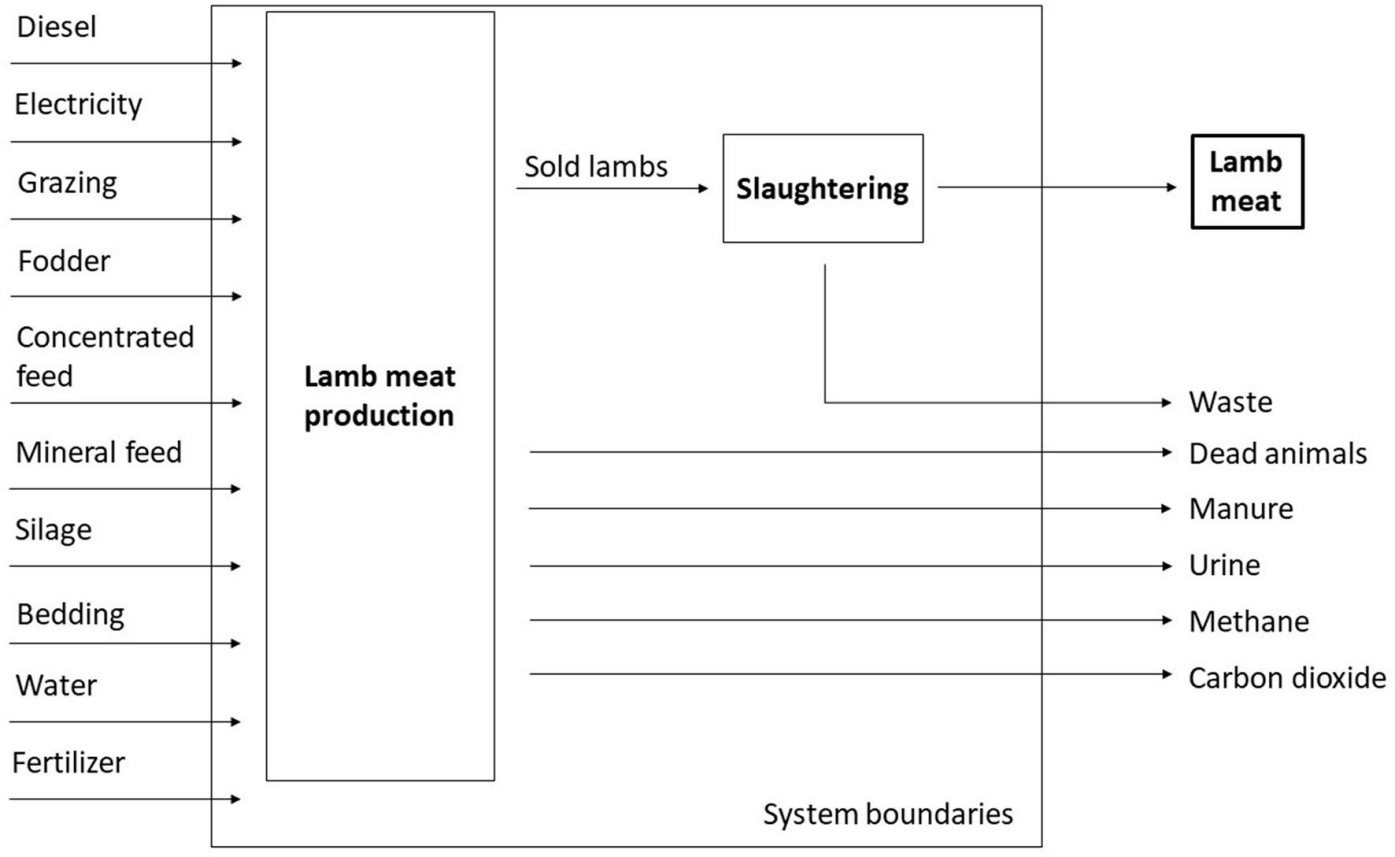
System boundaries with input and output flows.

The DIN EN ISO 14040 and DIN EN ISO 14044 standards provided guidelines for conducting a Life Cycle Assessment (LCA), and propose a four-step procedure for LCA. The first step is the definition of the goal and the scope. The inventory analysis is then drawn up, followed by an impact assessment, and, in conclusion, the results are to be interpreted (11, 12). The LCA was conducted accordingly using the GaBi software and databases. For the evaluation, the CML 2001 methodology, including the update in 2016 developed at Leiden University, was applied. It is an impact assessment method that limits uncertainties, restricting quantitative modeling to early steps in the production process. Results are grouped in midpoint categories (22). The scope was set to include the production and transport of fodder, bedding material, water, and fertilizer for the pastures as well as electricity and diesel fuel, the emissions from rumination, and the transport and processing of the lamb meat. Slaughtering was done by hand and therefore only the demand for electricity was relevant for the LCA. It is included in the overall electricity demand. The resulting system boundaries, including input and output lows, are shown in Figure 1.

The amount of fodder demand met by grazing was calculated via the necessary nutrition input through grass by subtracting the nutrition input from additional fodder that was purchased from the basal metabolic rate. The necessary nutrition input was then divided by nutrition content of grass (23). Amounts of manure, urine, water demand, emissions through rumination and respiration, and to the calorific value of the fodder were calculated in keeping with the literature (23). By-products like milk and wool were not accounted for, but all impacts of non-meat parts of the lamb were credited by waste incineration, including the production and provision of electric and thermal energy. The Life Cycle Inventory Analysis (LCIA) was based on primary data from the case study farms and supplemented by data from the literature and the GaBi database.

Since the yield of meat per kg liveweight varies greatly, depending on the form of livestock management and breed of sheep, the functional unit was set to 1 kg of lamb meat to provide for comparability among the different case studies. Given that the study focuses on meat production and the investigated farms breed mutton sheep, in contrast to wool or milk sheep, the non-meat parts were not counted as allocated secondary products, but the gain in electric and thermal energy from waste incineration was credited to the overall result.

The assessment focuses on the impact categories global warming potential (GWP), eutrophication potential (EP), and acidification potential (AP), with GWP representing the global effect and EP and AP standing for local effects. GWP is assessed in kg CO_2_ eq./kg lamb meat, EP in kg PO4 eq./kg lamb meat, and AP in kg SO2 eq./kg lamb meat.

#### Statistical Analysis for Hormone Evaluation

Statistical analysis was carried out using the program SPSS for Windows, version 23.0.

The Kolmogorov-Smirnov test for normality was employed to check whether the data followed a Gaussian distribution. As the normality was not verified for all results, different sets of parametric and non-parametric tests were used. The results were expressed as mean ± standard deviation.

To compare mean wool cortisol concentrations in the two different production systems and in the two data-collection periods, several tests were performed: the parametric unpaired and paired *t*-test, the one-way repeated measures ANOVA, the non-parametric Mann-Whitney U test, and the Friedman test with Wilcoxon post doc test. Statistical differences were considered to be significant at *P* < 0.05.

To compare mean wool cortisol concentrations in the two different productive systems, the parametric unpaired and paired *t*-test and the non-parametric Mann-Whitney U test were performed; although to compare mean wool cortisol concentration within the productive system during the 4-monthly time sampling (breeding time in 2018 and 2019), the one-way repeated measures ANOVA and the non-parametric alternative the Friedman test with Wilcoxon post doc test were performed. Statistical differences were considered significant at *P* < 0.05.

## Results and Discussion

### Chronic Stress Detection

Wool cortisol concentration of lambs enclosed in the study ranged from 1.59 to 41.65 pg/mg. The mean wool cortisol concentration in the two data collection periods ranged from 7.74 ± 3.30 to 14.87 ± 4.51 pg/mg for the two Winter (trial 1 and 3) for the Continental system, and from 10.84 ± 4.92 to 8.32 ± 6.84 pg/mg for the two Summer (trial 2 and 4) for the Alpine system.

**Figure 2:**
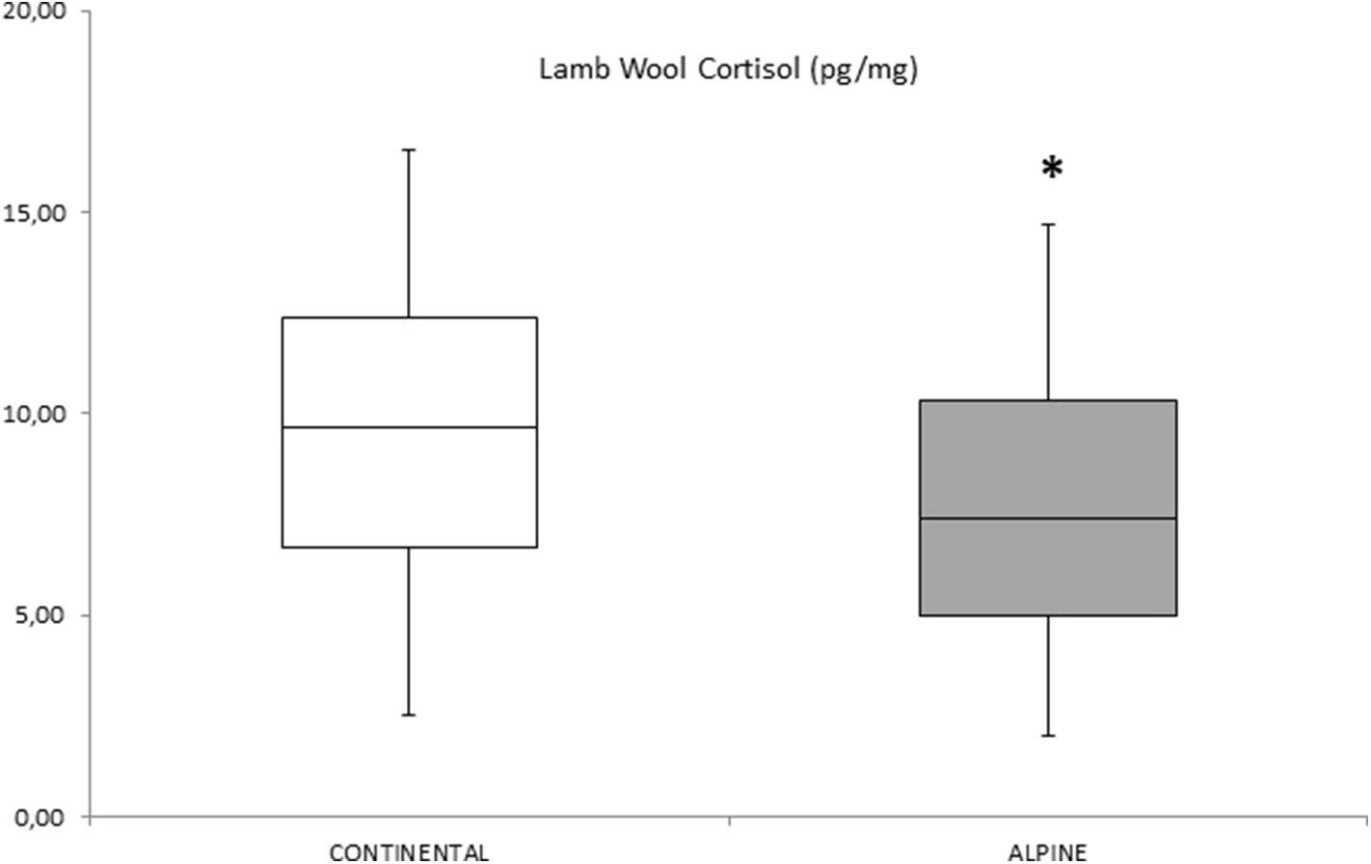
Wool cortisol concentration (pg/mg) in lamb reared in two different productive systems. Trials were repeated in 2018 and 2019 (twice each system). * Asterisk indicates significant difference (at least *p* < 0.02) between lamb wool cortisol concentration obtained from animals bred in continental vs. alpine system. The vertical bars denote the 25th to 75th and the whiskers the fifth to 95th percentile ranges.

The comparison of wool cortisol concentrations of lambs raised in the two different productive systems showed significant variations (*p* < 0.05). Wool cortisol concentration was significantly higher in lambs raised in the continental system than in lambs reared in the alpine mountain pasture (see Figure 2).

**Figure 3:**
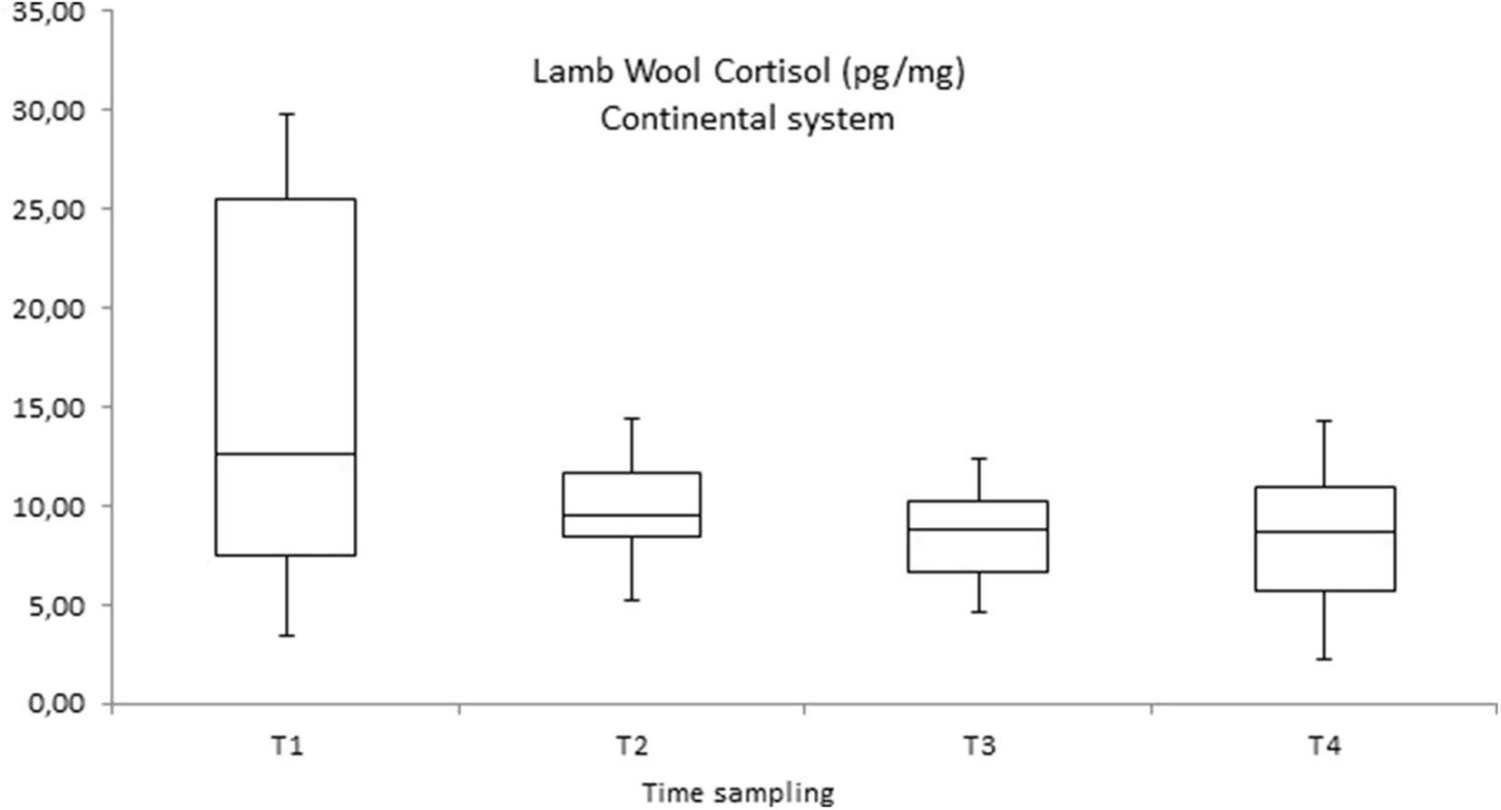
Wool cortisol concentration (pg/mg) measured in lambs in the continental system during the breeding periods in 2018 and 2019 (four months each year) sampling: T1 = first month of life; T2 = second month of life; T3 = third month of life; T4 = fourth month of life.

**Figure 4:**
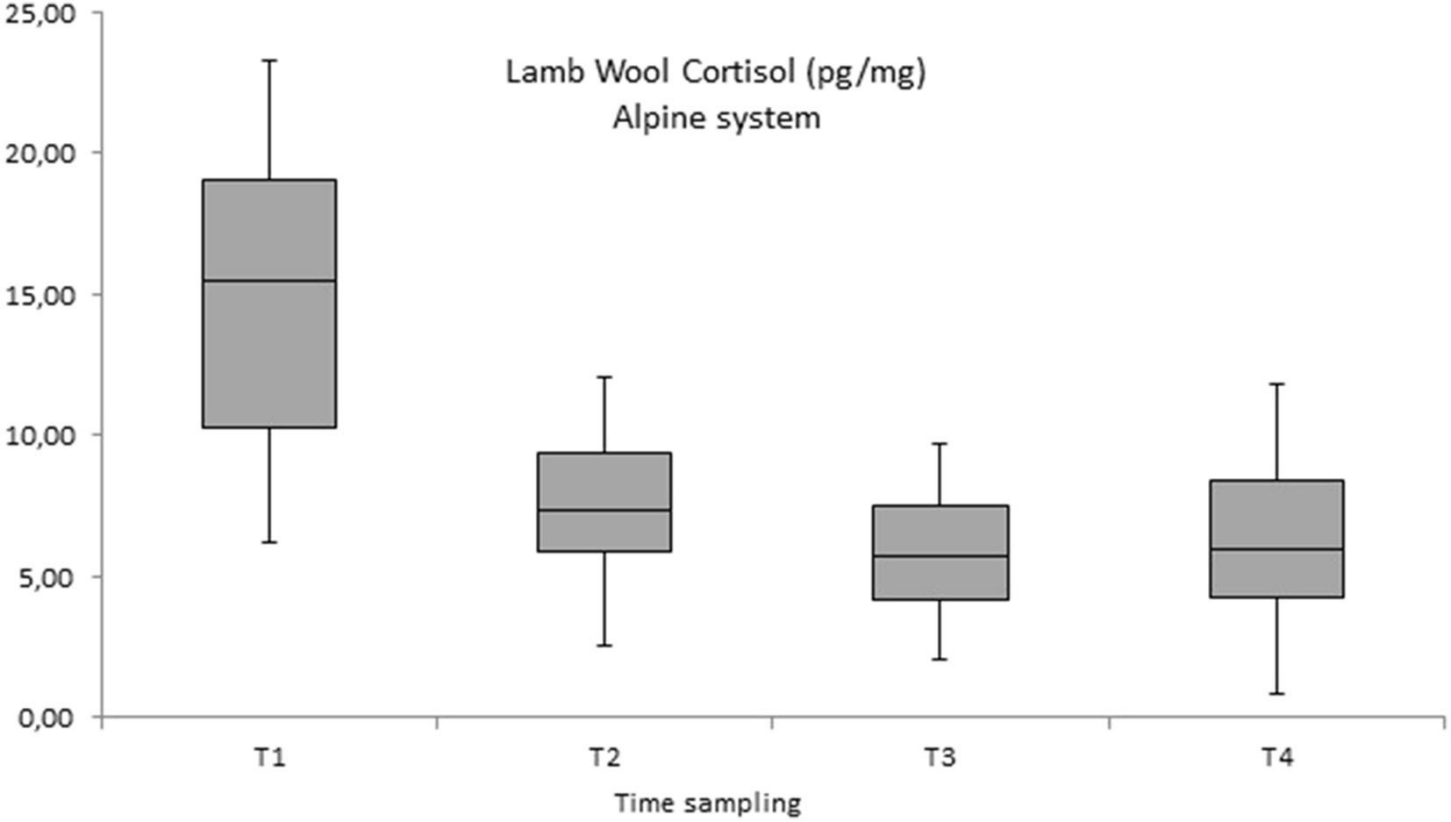
Wool cortisol concentration (pg/mg) measured in lambs in the alpine system during the breeding periods in 2018 and 2019 (four months each year) T1 = T1 = first month of life; T2 = second month of life; T3 = third month of life; T4 = fourth month of life.

Within the productive system the comparison between the four-monthly time of the wool collection showed high levels of cortisol in the first month of life (T1) with a significant decrease in values in the other three periods of wool collection (second, third, and fourth month of life—T2, T3, and T4) both in the continental and in the alpine system (see Figures 3, 4).

The absolute level in cortisol is comparable with previous studies (24, 25). Age, in fact, is a factor that influences the levels of cortisol in the hair, as at the moment of birth or at a young age it is higher and decreases with the passage of time, something that is also observed in other farming species.

### LCA Evaluation

**Figure 5:**
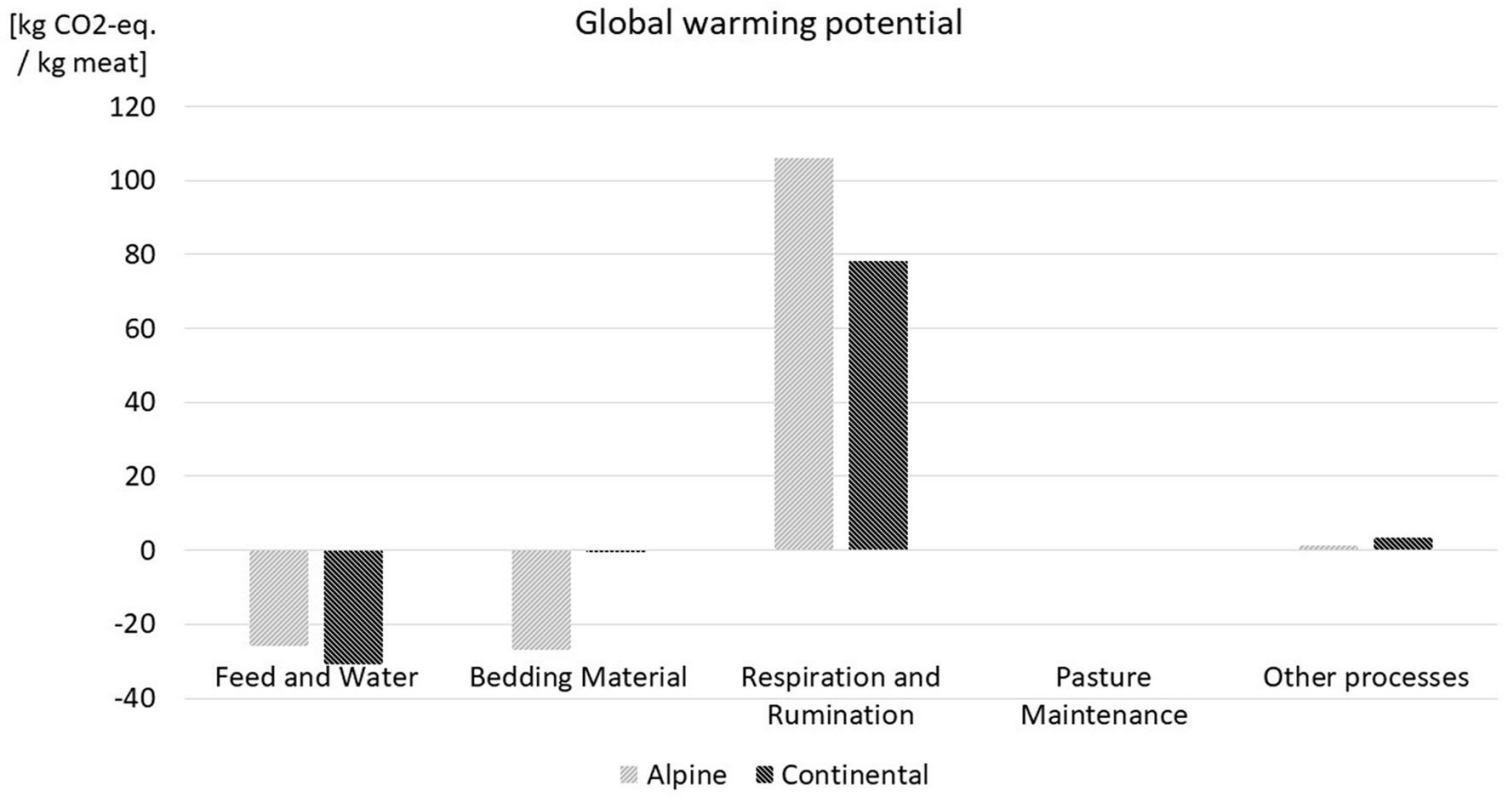
GWP results for both case studies.

**Figure 6:**
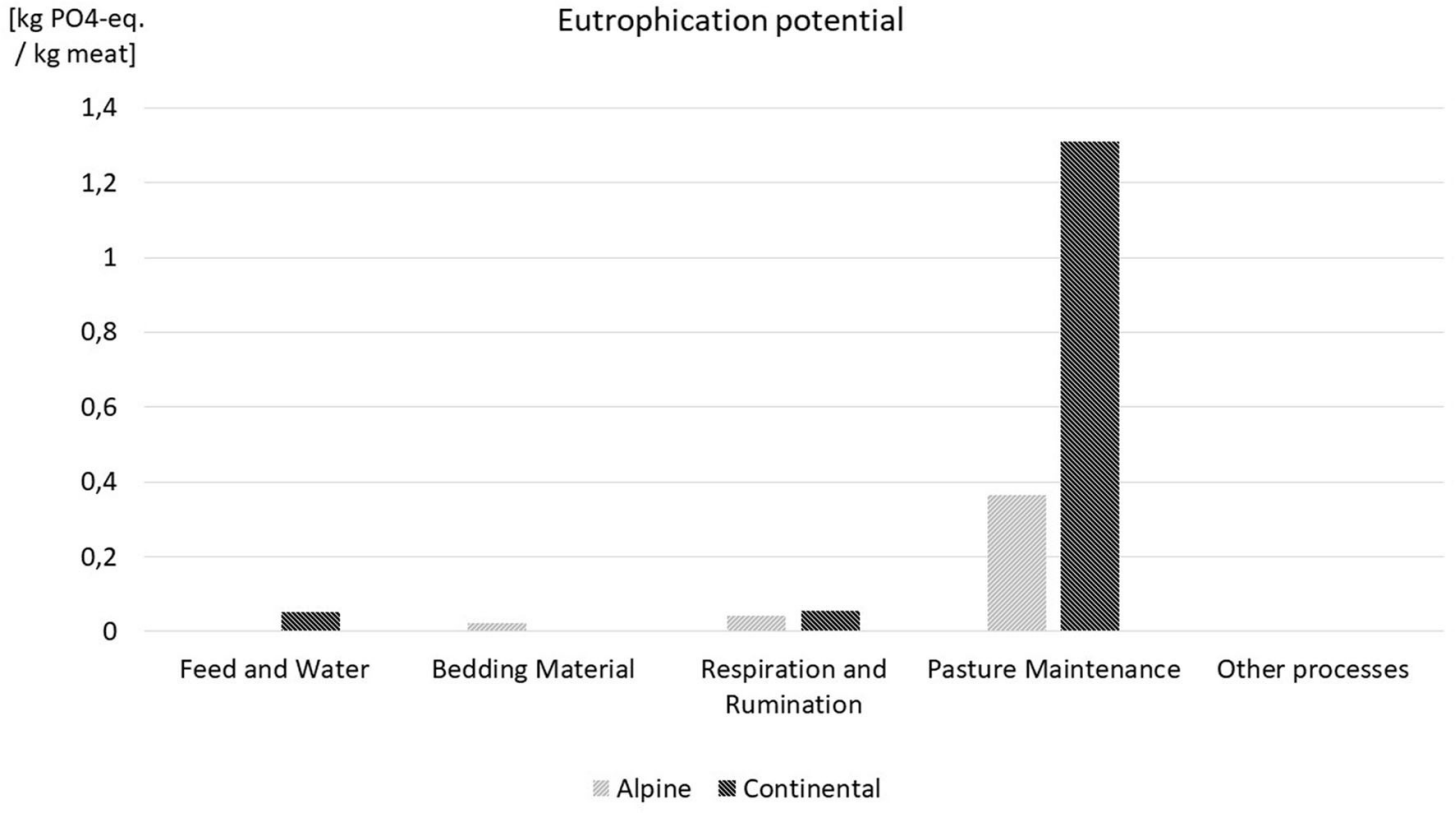
EP results for both case studies.

**Figure 7:**
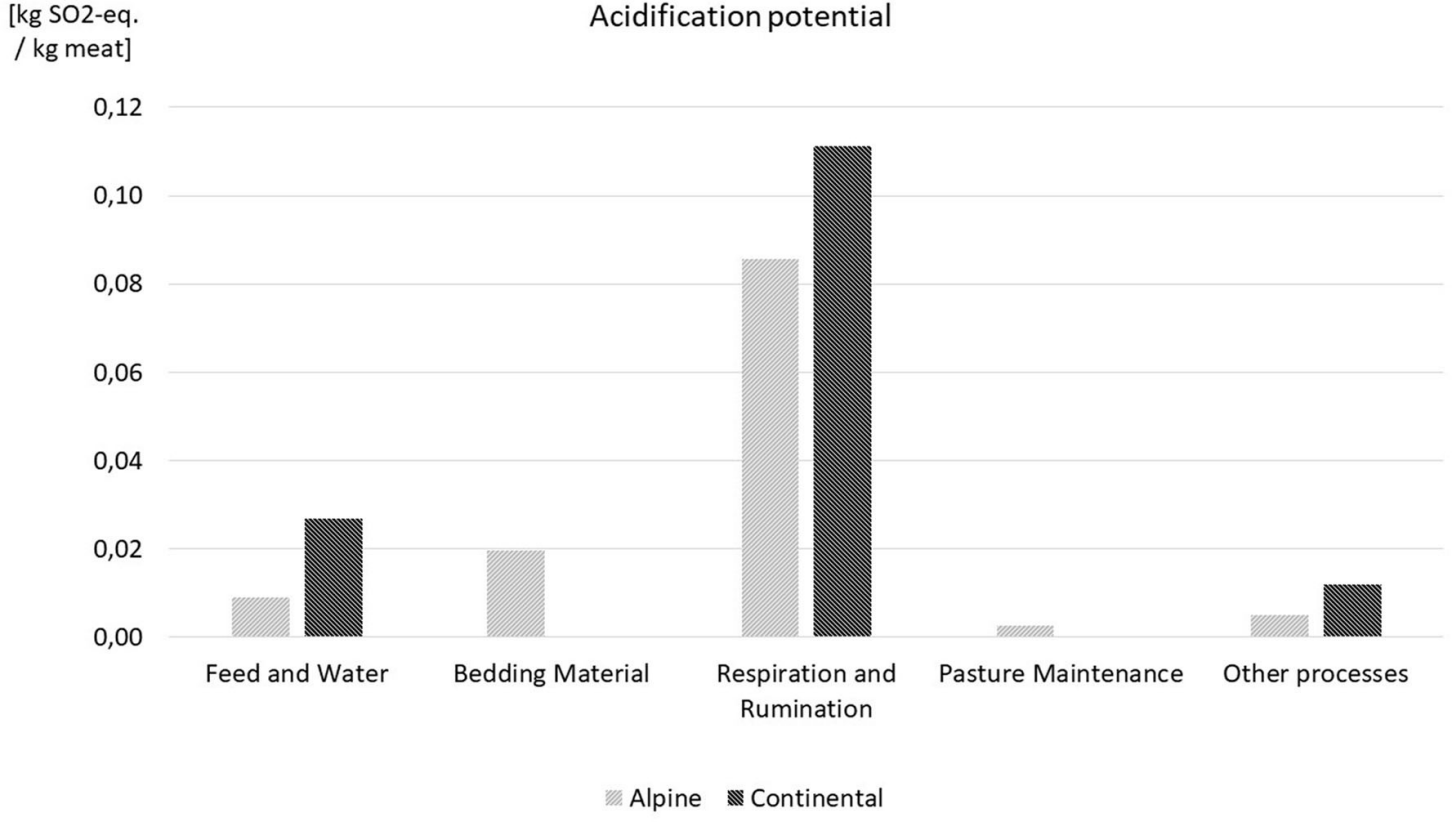
AP results for both case studies.

Table 1 shows the LCA results in detail; in Figures 5–7 a comparison for the most influential sets of processes of the life cycle of lamb meat is displayed. In Figures 5–7, processes with less influence on the overall results are clustered under the term “other processes,” including transport, fuel demand, waste incineration, and electricity and thermal energy processes. With respect to GWP and AP, respiration and rumination of the sheep, where the emissions of CO_2_ and CH4 are accounted for, have the highest share of impacts, while the discharges of manure, urine, and other fertilizing substances to the pastures represent the largest share of the eutrophication potential. The negative values in GWP of feed and water and bedding material are based on carbon dioxide fixation through plant growth. This fixation exceeds the impacts of usage of water and led to the overall negative values for feed and water. Also, the term fixation of carbon can be misleading here since the carbon is only bound in the plants, but further down the food chain it is partly emitted by the sheep and later on again partly emitted by the consumption of lamb meat. The fixation of carbon through plant growth is therefore a temporary occurrence. Since no thermal energy is used in lamb meat production, the negative amounts represent the credits in thermal energy from waste incineration.

**Table 1:** Overall LCA results for both case studies.

	**GWP**		**EP**		**AP**	
	**[kg CO**_****2****_ **eq/kg meat]**		**[kg PO**_****4****_ **eq/kg meat]**		**[kg SO**_****2****_ **eq/kg meat]**	
Case Study	Alpine	Continental	Alpine	Continental	Alpine	Continental
Feed and water	−2,58E+01	−3,07E+01	3,07E-03	5,01E-02	8,91E-03	2,69E-02
Bedding material	−2,70E+01	−9,74E-02	2,20E-02	7,95E-05	1,98E-02	7,13E-05
Respiration and rumination	1,06E+02	7,82E+01	4,10E-02	5,33E-02	8,57E-02	1,11E-01
Pasture maintenance	3,36E-01	1,50E-03	3,65E-01	1,31E+00	2,74E-03	1,14E-05
Transport	1,97E-02	1,02E-01	1,70E-05	8,21E-05	6,44E-05	3,11E-04
Fuel demand	3,11E-01	5,07E-01	9,61E-05	1,57E-04	1,21E-03	1,97E-03
Waste incineration	1,02E+00	1,95E+00	2,00E-04	3,83E-04	3,61E-03	6,90E-03
Electricity	7,73E-02	9,96E-01	1,35E-05	2,82E-04	1,87E-04	2,99E-03
Thermal energy	−1,13E-01	−2,69E-01	−1,02E-05	−2,42E-05	−5,81E-05	−1,38E-04
Total	5,50E+01	5,06E+01	4,32E-01	1,42E+00	1,22E-01	1,50E-01

The results show higher CO_2_ emissions for extensive production systems, which is due to the intensive farms' higher production efficiency and the larger meat gain on those farms. The factors contributing to those results include the use of faster-growing breeds, the higher nutrient content of concentrated feed and the consequently lower demand in fodder compared with grass and hay, in addition to the more frequent use of medication and medical surveillance. However, local impact categories, such as the eutrophication potential or the acidification potential, indicate that extensively managed sheep flocks have less of an effect on the surrounding flora and fauna that those raised in intensive systems.

In order to improve the results, primary data on respiration and rumination, manure and urine output and the individual carbon contents of the respective types of hay and grass could be collected. This would require chemical and biological analyses that exceed the scope of this study, which relied upon the literature and average data for these processes.

The results and findings derive from two case studies conducted over the course of 2 years. To validate the results and gain further knowledge on this topic, a continuation and extension of the study is recommended. The established models can be applied to similar sheep farms with comparable management practices.

## Discussion

In this study, we aimed to test a new LCA evaluation model in two different production systems of lamb that was slaughtered at 4 months of age. The two systems are the semi-intensive type, carried out in the Po Valley, in a continental region, and the semi-extensive type, practiced in alpine pastures in the northwestern area of Italy. We have also included an assessment of chronic stress, employing an index of animal welfare that can integrate the overall assessment of the supply chain through the analysis of cortisol accumulated in the animal's fleece every month during the breeding period.

A literature review showed that available LCA studies of lamb meat production in the EU show results that vary considerably from ca. 15 kg CO_2_ eq./kg meat up to ca. 57 kg CO_2_ eq./kg meat. A median of 32.7 kg CO_2_ eq./kg meat was calculated. The production systems in the EU show the highest emissions per kg meat worldwide since the farms are managed rather extensively (26). The main factors for the large range in GWP are the allocation of secondary products and credits in GWP due to landscape management and ecosystem services among other things. In this study, no secondary products were accounted for, and credits were only given for the carbon sequestration through plant growth for the production of fodder, and the energy gain through waste incineration of meat and production residues. Therefore, the results of the GWP from this study are to be found in the upper half of the range given in the literature review.

Similar trends have also been found in dairy sheep production systems. Greenhouse gas emissions vary from 1.77 to 4.09 Kg CO_2_ eq./kg of milk; the lowest values correspond to the most intensively run farms and the highest values to the most extensively run, less productive farms. Enteric fermentation is a consequence of feeding at pasture. Enteric fermentation reaches its maximum value (52.22 % of the total emissions) in the most extensive farms. This confirms the proposition regarding the higher emission values of extensive farming systems for GWP.

It is, however, well known that extensive and well-managed livestock farming systems tend to achieve a balance between production and conservation thanks to adjustments between the stocking rate and natural resources. Favorable effects resulting from pastoral practice (for example, biodiversity) are of fundamental importance to the health of the surrounding ecosystems (27). In addition, extensive grazing is also a major source of nutrients for the soil, as animal excreta contribute to the improvement of the soil structure by increasing its organic matter content and hereby maintaining an adequate plant cover (28, 29). Sheep farming is one of the most important livestock activities in these pastoral areas, as it is well suited to the climate and the type of local fodder (natural pasture) available.

The assessments for EP and AP, for instance, are indicators for these factors and impacts. In our case study, the significantly lower results of EP and AP of the extensive case study in the alpine bioregion point to the value of pastoral-based livestock farming systems.

Together with these considerations, we also report the data on the concentration of cortisol, which is an index of stress assessment (30) and is to a significant degree less common in the extensive breeding system, despite the various activities and experiences that the animals go through, and has an effect on this parameter (daily motor activity along high altitude slopes, sudden climatic changes, presence of predators). We evaluated the production and welfare parameters of lambs from birth to slaughter. In contrast to saliva and blood cortisol, studies in other species have shown that hair cortisol is a proxy measure to the total retrospective activity of the HPA-axis over weeks or even months (24, 25, 31). Our data show clearly that the hair cortisol was less accumulated during the extensive breeding despite the various activities and experiences that the animals undergo, which affects this parameter (daily motor activity along high altitude slopes, sudden climatic changes, and presence of predators). This parameter indicates a better adaptability to the environment, provides information that is increasingly being considered by the final consumer (27, 28), and has been reported to have some consequences on the quality of the meat product with respect to lamb (32) and pork (33, 34).

In fact, our opinion is evaluation of the breeding system has to increasingly take into account the potentially higher “willingness to pay,” on the side of consumers, for welfare-friendly meat, which may potentially increase profits even further. Previous studies have shown that consumers are willing to make an extra effort to buy animal welfare-friendly products even if this means paying more for goods or changing where they shop (35, 36).

## Conclusion

This research emphasizes that the evaluation of an animal supply chain has yet to be evaluated very carefully, as there are many aspects that must be considered in accordance with the various interactions with the local environment. This applies in particular to the sheep-farming sector, where interaction with marginal but important local environments, from an ecological point of view, must be reconsidered in an overall assessment of the production chain's ecological sustainability.

The two breeding systems are subject to further analysis for a more complete assessment of the stress state to which the animals are subjected, but we believe it is interesting to show these first data, in particular to open a discussion regarding the parameters that should be included for a holistic sustainability evaluation. In an overall evaluation of the supply chain, for example, the assessment of the quality and hygienic safety of the meat product must also be included.

The results of this case study show the quantifiable positive effects that grazing sheep and extensive traditional farming practices have on their local ecosystems and on animal welfare without ignoring the environmental advantages of intensification and more efficient production. There is, however, a need for further research to broaden the geographic range of the case studies and collect primary data from various farming systems in different locations throughout the world; in addition, seasonal and annual variations in production should be taken into account by collecting data over longer periods of time to provide larger data sets for the evaluation of the effects being studied.

## Data Availability Statement

All datasets presented in this study are included in the article/Supplementary Material.

## Ethics Statement

Ethical review and approval was not required for the animal study because the research was conducted at sheep farms and the data collected were either business data or derived from hair samples, that were taken from hair, that was shaved off within the normal practices of sheep management. Written informed consent was obtained from the owners for the participation of their animals in this study.

## Author Contributions

AG provided the development of the LCA model and the following evaluation and assessment of this model. IV and GP were responsible for the animal management and sample collection. SM and EM conducted the hormone and animal behavioral analysis. LB lead the critical discussion and evaluation of the animal welfare analysis. MB was responsible for the experimental design, critical discussion and manuscript writing and also provided guidance in the preparation of this paper. All authors contributed to the article and approved the submitted version.

## Conflict of Interest

The authors declare that the research was conducted in the absence of any commercial or financial relationships that could be construed as a potential conflict of interest.
