# Supplementary figures and images for: A New Approach to LCA Evaluation of Lamb Meat Production in Two Different Breeding Systems in Northern Italy
Source: Front Vet Sci. 2020 Sep 28;7:651. doi: 10.3389/fvets.2020.00651 (PMC7549392; doi:10.3389/fvets.2020.00651)

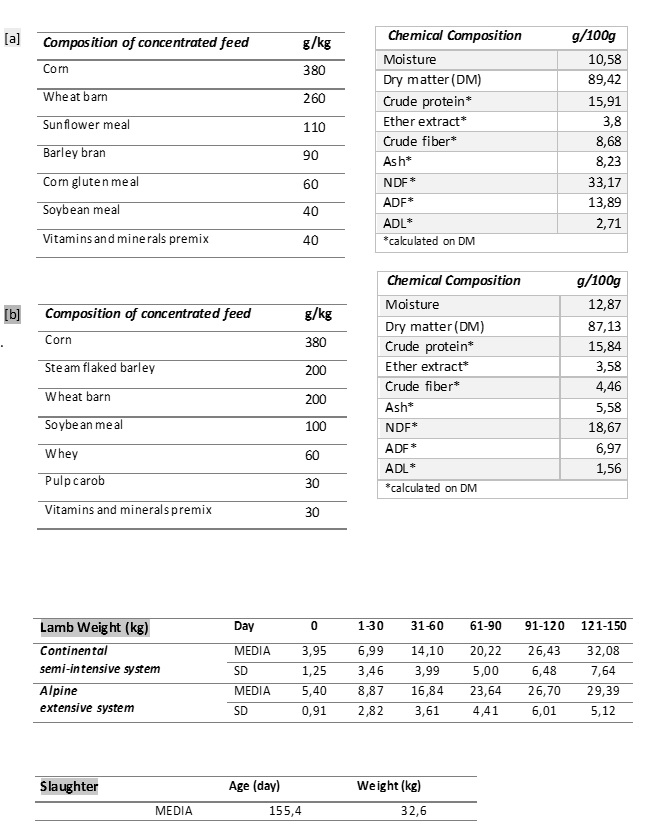

Supplement: Supplementary file 1 [file Image_1.JPEG]
